# Molecular Characterization of Chikungunya Virus, Philippines, 2011–2013

**DOI:** 10.3201/eid2205.151268

**Published:** 2016-05

**Authors:** Ava Kristy Sy, Mariko Saito-Obata, Inez Andrea Medado, Kentaro Tohma, Clyde Dapat, Edelwisa Segubre-Mercado, Amado Tandoc, Socorro Lupisan, Hitoshi Oshitani

**Affiliations:** Research Institute for Tropical Medicine, Muntinlupa City, Philippines (A.K. Sy, I.A. Medado, E. Segubre-Mercado, A. Tandoc III, S. Lupisan);; Tohoku University Graduate School of Medicine, Sendai, Japan (M. Saito-Obata, K. Tohma, C. Dapat, H. Oshitani);; Tohoku–Research Institute for Tropical Medicine Collaborating Research Center on Emerging and Reemerging Infectious Diseases, Muntinlupa City (M. Saito-Obata, C. Dapat, H. Oshitani)

**Keywords:** Chikungunya virus, molecular epidemiology, genotype, outbreak, Philippines, viruses, vector-borne infections, mosquitoes

## Abstract

During 2011–2013, a nationwide outbreak of chikungunya virus infection occurred in the Philippines. The Asian genotype was identified as the predominant genotype; sporadic cases of the East/Central/South African genotype were detected in Mindanao. Further monitoring is needed to define the transmission pattern of this virus in the Philippines.

Chikungunya fever is a mosquitoborne infection that causes large outbreaks mainly in tropical and subtropical countries. The causative agent is chikungunya virus (CHIKV), an enveloped, single-stranded positive-sense RNA virus (family *Togaviridae*, genus *Alphavirus*). Phylogenetic analysis of the E1 gene of CHIKV revealed 3 genotypes: West African, East/Central/South African (ECSA), and Asian (*1*). In 2005, large outbreaks occurred in the islands in the Indian Ocean and India that were caused by the Indian Ocean lineage (IOL) virus, which newly emerged from the ECSA genotype (*2*). More recently, the emergence and potential spread of ECSA and Asian genotypes in the Americas have been major public health concerns in the region. (*3*).

In the Philippines, CHIKV was first isolated in 1965 (*4*). Since then, sporadic cases have been reported, including those among US Peace Corps volunteers stationed in the islands of Mindanao, Cebu, and Masbate in 1986 (*5*), and a local community outbreak in Cavite, Luzon Island, was reported in 1996 (*6*). However, the first nationwide CHIKV outbreak was identified starting in 2011 (Philippines Department of Health, unpub. data). Previous studies of chikungunya fever in the Philippines focused on clinical and serologic analyses (*7*), and recent reports on the molecular surveillance of CHIKV in the country were limited and analyzed only samples collected in 2012 (*8*,*9*). We conducted genetic analysis to characterize recent CHIKV infections that caused a large nationwide outbreak in the Philippines during 2011–2013.

## The Study

Serum samples were collected through the chikungunya fever surveillance under the Philippine Integrated Disease Surveillance and Response of the Department of Health Epidemiology Bureau from different provinces in the Philippines during 2011–2013. Samples were collected from patients suspected to have chikungunya fever manifesting with symptoms such as fever, rash, and arthralgia. Samples were sent to the Research Institute for Tropical Medicine, which serves as the National Reference Laboratory for Dengue and Other Arboviruses. We screened serum samples were screened by using CHIKV IgM-capture ELISA (NovaLisa, NovaTec Immundiagnostica GmbH, Dietzenbach, Germany). We extracted viral RNA using the QIAamp Viral RNA kit (QIAGEN, Hilden, Germany) according to the manufacturer’s instructions and amplified the partial E1 gene using 1-step reverse transcription PCR followed by direct Sanger sequencing with primers as previously described (*10*,*11*). We conducted phylogenetic analysis using the maximum-likelihood method as implemented in MEGA 6 software (http://www.megasoftware.net/). Molecular clock analysis and Bayes factor calculation were performed using BEAST software 1.8.0 (http://beast.bio.ed.ac.uk/) to select the best migration event model of CHIKV among countries. Bayes factor analysis was used to test phylogeographic hypothesis whether posterior migration rate between locations in whole evolutionary history was significantly higher than the expected prior migration rate, assuming truncated Poisson probability (*12*).

A total of 5,729 serum samples were collected from persons suspected to have chikungunya within 5 days after symptom onset. Fever, rash, and arthralgia were the most common symptoms (53%, 47%, and 34% of patients, respectively). Of the 5,729 serum samples, 2,891 were IgM positive by ELISA. We conducted reverse transcription PCR on 382 representative samples among IgM-negative patients in accordance with the CHIKV outbreak surveillance strategy of the Philippines, of which 131 samples tested positive. Partial E1 gene sequence (733 nt) was obtained from 31 samples. Sequences were submitted to GenBank (accession nos. LC064714–LC064744).

**Figure 1:**
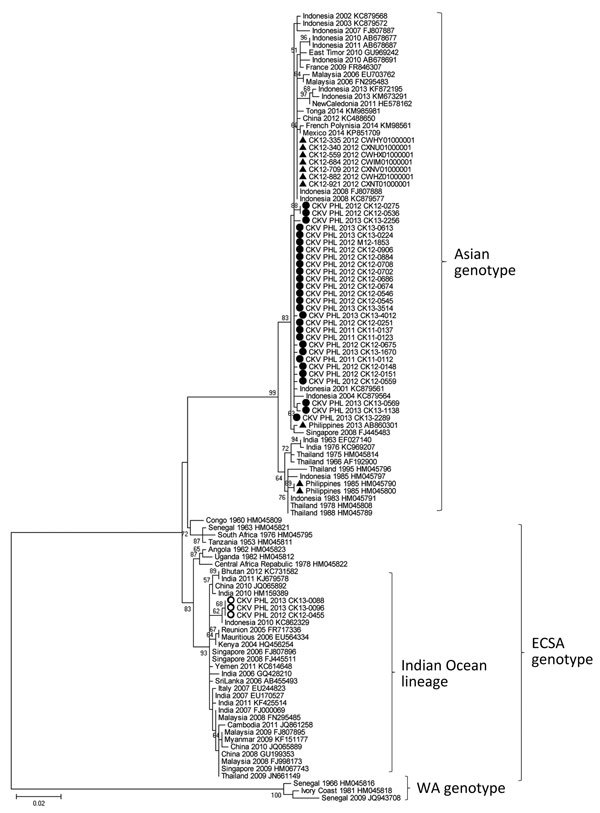
Phylogenetic analysis of partial (733 nt) E1 gene of 31 CHIKVs detected in the Philippines in this study during 2011–2013 compared with 77 global strains. The tree was constructed using maximum-likelihood method with the Kimura 2-parameter model and 1,000 bootstrap replications. Bootstrap values >50% are indicated on the branches of the tree. Black circles indicate Asian genotypes; open circles indicate ECSA genotypes analyzed in this study; triangles indicate reference strains collected in the Philippines. CHIKV, chikungunya virus; ECSA, East/Central/South African, WA, West African. Scale bar indicates nucleotide substitutions per site.

Phylogenetic analysis identified 28 Asian genotype viruses and 3 ECSA genotype viruses (Figure 1). Sequence analysis of E1 gene showed >99% nt similarity among 28 Asian genotype viruses (data not shown). Bayes factor analysis for the migration events also showed that migration might have occurred between Indonesia and Philippines with high probability (Table). CHIKV-positive patients were found only in Mindanao Island in 2011 but were detected in other parts of the country in 2012 and 2013. All CHIKV detected in parts of the country other than Mindanao were identified as Asian genotype.

**Table:** Bayes factor of migration events of Asian genotype of chikungunya virus based on 65 cases, Philippines, 2011–2013*

Bayes factor	Locations
68.8	Philippines, Indonesia
68.0	India, Thailand
54.2	Indonesia, New Caledonia
25.6	French Polynesia, Mexico
22.0	France, Indonesia
19.5	Indonesia, Malaysia
11.3	East Timor, Indonesia
7.6	Indonesia, Singapore
6.8	Indonesia, Thailand
3.4	China, Tonga
3.3	China, Indonesia
2.3	East Timor, France
2.3	Philippines, Thailand
2.2	Mexico, Tonga
2.1	French Polynesia, Tonga
2.1	China, French Polynesia
2.0	Philippines, Tonga
1.9	China, Mexico
1.7	China, Philippines
1.3	Malaysia, Singapore

*The migrations of Asian genotype viruses between countries were determined by using Bayes factor analysis. Bayes factor value >5 is considered a significant migration.

**Figure 2:**
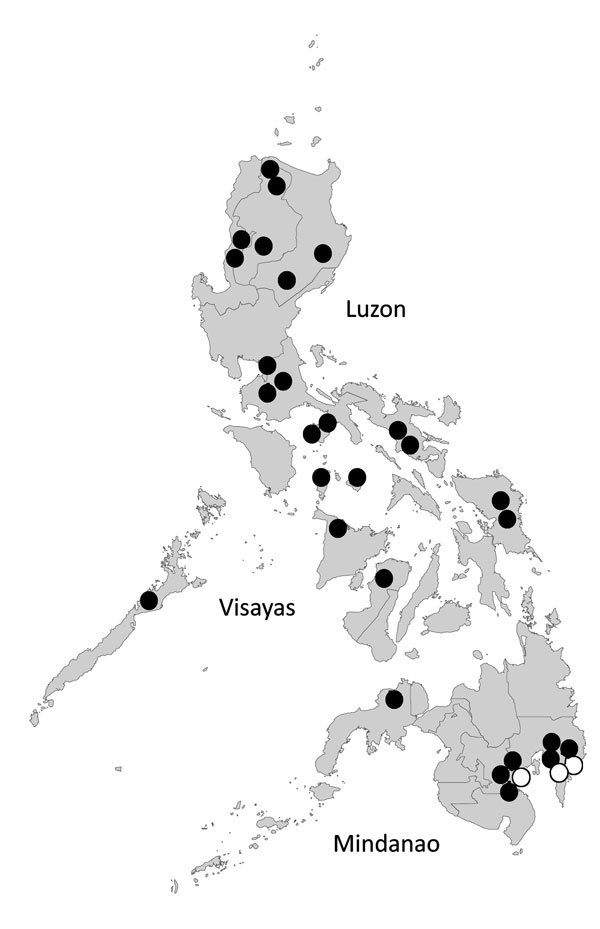
Geographic distribution of CHIKV genotypes in the Philippines. The location of samples collected in this study are indicated by circles; 1 circle represents 1 sample. Black circles indicate Asian genotype; white (open) circles indicate East/Central/South African genotype. CHIKV, chikungunya virus.

The 3 ECSA genotype viruses were detected in the Davao Del Sur and Davao Oriental in Mindanao Island in 2012 and 2013, and all were clustered into IOL (Figure 1,2). This finding indicates that the 2 genotypes co-circulated in the island during the outbreak. ECSA genotype viruses in the Philippines were closely related to the Indonesian viruses (GenBank accession no. KC862329) detected in 2010. Sequencing analysis showed that all Philippine ECSA viruses possess the alanine to valine substitution (A226V) in the E1 gene (Technical Appendix Tables 1, 2).

## Conclusions

The recent large CHIKV outbreaks in other Asian countries were caused mainly by the IOL of ECSA genotype (*2*). However, the 2011–2013 outbreak in the Philippines was caused mainly by the Asian genotype; the reason that this large outbreak was caused by this genotype is unknown. Previous reports have confirmed the reemergence of Asian genotype viruses in the Philippines (*8**,**9*). In this study, phylogenetic analysis and Bayes factor calculation showed that the Philippines viruses were closely related to Indonesian viruses, which might explain why the outbreak started in southern Mindanao, near Indonesia. Although sequence data of CHIKV in the database are not enough to identify the exact origin of the virus, we tried to estimate when CHIKV was introduced into the Philippines with molecular clock analysis using the dataset of Asian genotype viruses (Technical Appendix Figures 1, 2). The results suggested that circulating Asian genotype viruses in the Philippines were introduced from Indonesia before 2010.

We also detected IOL of ECSA genotype, which possesses the E1-A226V substitution, and its co-circulation with the Asian genotype in the Philippines. However, the geographic distribution of ECSA genotype in 2012 and 2013 was limited to southern provinces in Mindanao. After the large outbreak in the Indian Ocean region in 2005, IOL of ECSA genotype rapidly spread to Asian countries and then co-circulated with the endemic Asian viruses, then eventually became the predominant genotype (*13*). However, this circulation pattern differs from what we observed in the Philippines. Until the early 2010s, most of the viruses circulating in the Philippines and Indonesia were still Asian genotype, and ECSA genotype viruses had been reported in West Kalimantan, Indonesia, in 2011 (*14*) and in the Philippines in 2012 several years after the large outbreak in the Indian Ocean region (*2*). The movement of persons near the border of these countries might play a key role in CHIKV transmission.

As part of the national vector control program, surveillance of *Aedes aegypti* and *Ae. albopictus* mosquitoes has been conducted in several areas in the Philippines. A previous report showed that the proportion of these 2 mosquito species was almost the same in Metro Manila (*15*). If the proportion of *Ae. albopictus* mosquitoes increases, the ESCA genotype virus with A226V mutation could spread more rapidly in the country. Thus, monitoring the spread of ECSA genotype viruses and the proportion of the *Aedes* mosquitoes in the Philippines is important.

We have demonstrated that the Asian genotype CHIKV, which is closely related to the Indonesian viruses, was identified in Mindanao in 2011 and spread to other regions in 2012 and 2013. Like the Asian genotype, ECSA genotype virus was first detected in Mindanao in 2012. Mindanao might play a key role for the introduction of the CHIKV into the Philippines. Further monitoring is necessary to define the transmission pattern of CHIKV, including cross-border transmission.

Technical Appendix Markov jump density of viral migrations of CHIKV over time between Philippines and Indonesia; newly sequenced CHIKV in this study; maximum clade credibility tree of Asian genotype CHIKV; and reference strains obtained from GenBank used in this study.
