# Supplementary material for: Molecular Characterization of Chikungunya Virus, Philippines, 2011–2013
Source: Emerg Infect Dis. 2016 May;22(5):887–90. doi: 10.3201/eid2205.151268 (PMC4861512; doi:10.3201/eid2205.151268)
Supplement: Technical Appendix — Markov jump density of viral migrations of CHIKV over time between Philippines and Indonesia; newly sequenced CHIKV in this study; maximum clade credibility tree of Asian genotype CHIKV; and reference strains obtained from GenBank used in this study. [file 15-1268-Techapp-s1.pdf]

Article DOI: http://dx.doi.org/10.3201/eid2205.151268

Molecular Characterization of Chikungunya Virus, Philippines, 2011–2013

Technical Appendix

Technical Appendix Table 1. Chikungunya viruses newly sequenced in this study, Philippines, 2011–2013, n = 31

Strain name	GenBank accession no.	Genotype	Amino acid at position 226	Year collected	Month collected	Country	Region	Province
CKV_PHL_2013_CK13–0613	LC064714	Asian	Alanine	2013	July	Philippines	CAR	Ifugao
CKV_PHL_2013_CK13–2256	LC064715	Asian	Alanine	2013	Apr	Philippines	CAR	Apayao
CKV_PHL_2012_CK12–0151	LC064716	Asian	Alanine	2012	Nov	Philippines	NCR	NCR
CKV_PHL_2013_CK13–1138	LC064717	Asian	Alanine	2013	Nov	Philippines	1	LaUnion
CKV_PHL_2013_CK13–2289	LC064718	Asian	Alanine	2013	Aug	Philippines	1	Ilocos Sur
CKV_PHL_2012_CK12–0559	LC064728	Asian	Alanine	2012	Oct	Philippines	2	Isabela
CKV_PHL_2013_CK13–1670	LC064729	Asian	Alanine	2013	July	Philippines	2	Quirino
CKV_PHL_2013_CK13–3514	LC064730	Asian	Alanine	2013	Aug	Philippines	2	CagayanValley
CKV_PHL_2012_CK12–0148	LC064731	Asian	Alanine	2012	Aug	Philippines	4A	Laguna
CKV_PHL_2012_CK12–0251	LC064732	Asian	Alanine	2012	Aug	Philippines	4A	Laguna
CKV_PHL_2012_CK12–0545	LC064733	Asian	Alanine	2012	Oct	Philippines	4B	Romblon
CKV_PHL_2012_CK12–0546	LC064734	Asian	Alanine	2012	Dec	Philippines	4B	Romblon
CKV_PHL_2012_CK12–0702	LC064735	Asian	Alanine	2012	Nov	Philippines	4B	Marinduque
CKV_.PHL_2012_CK12–0708	LC064736	Asian	Alanine	2012	Nov	Philippines	4B	Marinduque

Strain name	GenBank accession no.	Genotype	Amino acid at position 226	Year collected	Month collected	Country	Region	Province
CKV_PHL_2012_M12–1853	LC064737	Asian	Alanine	2012	July	Philippines	4B	Palawan
CKV_PHL_2012_CK12–0674	LC064738	Asian	Alanine	2012	Oct	Philippines	5	Albay
CKV_PHL_2012_CK12–0675	LC064739	Asian	Alanine	2012	Jun	Philippines	5	Albay
CKV_PHL_2013_CK13–0569	LC064740	Asian	Alanine	2013	July	Philippines	6	Negros Occidental
CKV_PHL_2013_CK13–4012	LC064741	Asian	Alanine	2013	Nov	Philippines	6	Aklan
CKV_PHL_2012_CK12–0884	LC064742	Asian	Alanine	2012	Dec	Philippines	8	Samar
CKV_PHL_2012_CK12–0906	LC064743	Asian	Alanine	2012	Feb	Philippines	8	Samar
CKV_PHL_2013_CK13–0224	LC064744	Asian	Alanine	2013	July	Philippines	9	Zamboanga Del Norte
CKV_PHL_2011_CK11–0112	LC064719	Asian	Alanine	2011	Oct	Philippines	11	Davao Del Sur
CKV_PHL_2011_CK11–0123	LC064720	Asian	Alanine	2011	Oct	Philippines	11	Davao Del Sur
CKV_PHL_2011_CK11–0137	LC064721	Asian	Alanine	2011	Sept	Philippines	11	Davao Del Sur
CKV_PHL_2012_CK12–0275	LC064722	Asian	Alanine	2012	Oct	Philippines	11	CompostellaValley
CKV_PHL_2012_CK12–0536	LC064724	Asian	Alanine	2012	Nov	Philippines	11	CompostellaValley
CKV_PHL_2012_CK12–0686	LC064725	Asian	Alanine	2012	Oct	Philippines	11	CompostellaValley
CKV_PHL_2012_CK12–0455	LC064723	ECSA	Valine	2012	Jan	Philippines	11	Davao Del Sur
CKV_PHL_2013_CK13–0088	LC064726	ECSA	Valine	2013	Jan	Philippines	11	Davao Oriental
CKV_PHL_2013_CK13–0096	LC064727	ECSA	Valine	2013	May	Philippines	11	Davao Oriental

*CAR, Cordillera Administrative Region; ECSA, East/Central/South African; NCR, National Capital Region.

Technical Appendix Table 2. Reference strains obtained from GenBank used in this study,* n = 77

Country	Year collected	GenBank accession no.	Genotype
Angola	1962	HM045823	ECSA
Bhutan	2012	KC731582	ECSA
Cambodia	2011	JQ861258	ECSA
Central African Republic	1978	HM045822	ECSA
China	2008	GU199353	ECSA
China	2010	JQ065889	ECSA
China	2010	JQ065892	ECSA
China	2012	KC488650	Asian
Congo	1960	HM045809	ECSA
East Timor	2010	GU969242	Asian
France	2009	FR846307	Asian
French Polynesia	2014	KM98561	Asian
India	1963	EF027140	Asian
India	1976	KC969207	Asian
India	2006	GQ428210	ECSA
India	2007	EU170527	ECSA
India	2007	FJ000069	ECSA
India	2010	HM159389	ECSA
India	2011	KF425514	ECSA
India	2011	KJ679578	ECSA
Indonesia	1983	HM045791	Asian
Indonesia	1985	HM045797	Asian
Indonesia	2001	KC879561	Asian
Indonesia	2002	KC879568	Asian
Indonesia	2003	KC879572	Asian
Indonesia	2004	KC879564	Asian
Indonesia	2007	FJ807887	Asian
Indonesia	2008	FJ807888	Asian
Indonesia	2008	KC879577	Asian
Indonesia	2010	AB678677	Asian
Indonesia	2010	AB678691	Asian
Indonesia	2010	KC862329	ECSA
Indonesia	2011	AB678687	Asian
Indonesia	2013	KF872195	Asian
Indonesia	2013	KM673291	Asian
Italy	2007	EU244823	ECSA

Country	Year collected	GenBank accession no.	Genotype
IvoryCoast	1981	HM045818	WA
Kenya	2004	HQ456254	ECSA
Reunion	2005	FR717336	ECSA
Malaysia	2006	EU703762	Asian
Malaysia	2006	FN295483	Asian
Malaysia	2008	FJ998173	ECSA
Malaysia	2008	FN295485	ECSA
Malaysia	2009	FJ807895	ECSA
Mauritious	2006	EU564334	ECSA
Mexico	2014	KP851709	Asian
Myanmar	2009	KF151177	ECSA
New Caledonia	2011	HE578162	Asian
Philippines	1985	HM045790	Asian
Philippines	1985	HM045800	Asian
Philippines	2012	CWHY01000001	Asian
Philippines	2012	CXNU01000001	Asian
Philippines	2012	CWHX01000001	Asian
Philippines	2012	CWIM01000001	Asian
Philippines	2012	CXNV01000001	Asian
Philippines	2012	CWHZ01000001	Asian
Philippines	2012	CXNT01000001	Asian
Philippines	2013	AB860301	Asian
Senegal	1963	HM045821	ECSA
Senegal	1966	HM045816	WA
Senegal	2009	JQ943708	WA
Singapore	2006	FJ807896	ECSA
Singapore	2008	FJ445483	Asian
Singapore	2008	FJ445511	ECSA
Singapore	2009	HM067743	ECSA
South Africa	1976	HM045795	ECSA
Sri Lanka	2006	AB455493	ECSA
Tanzania	1953	HM045811	ECSA
Thailand	1966	AF192900	Asian
Thailand	1975	HM045814	Asian
Thailand	1978	HM045808	Asian
Thailand	1988	HM045789	ECSA
Thailand	1995	HM045796	ECSA

Country	Year collected	GenBank accession no.	Genotype
Thailand	2009	JN661149	ECSA
Tonga	2014	KM985981	Asian
Uganda	1982	HM045812	ECSA
Yemen	2011	KC614648	ECSA

*ECSA, East/Central/South African; WA, West African.

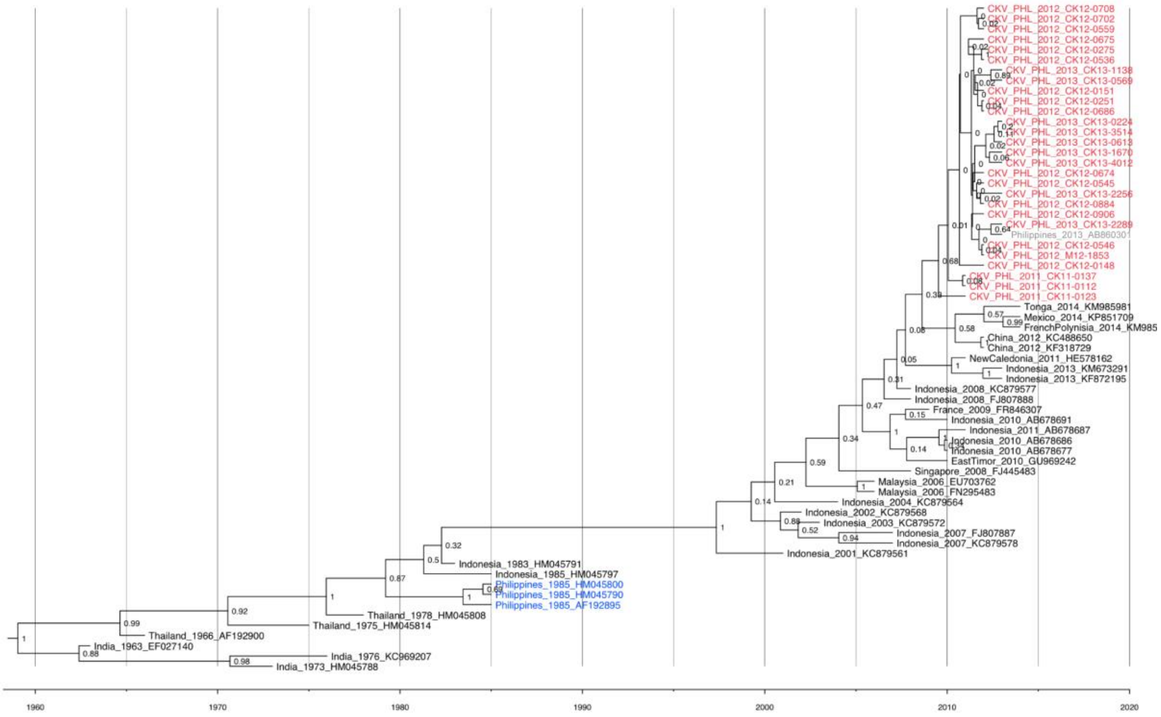

Technical Appendix Figure 1. Maximum clade credibility (MCC) tree of Asian genotype of chikungunya virus. MCC tree of Asian genotype was inferred by molecular clock analysis. Branch lengths are temporally scaled, and the x-axis shows the time unit (year). Red indicates Philippine viruses analyzed in this study; blue indicates Philippine strains in the 1980s obtained from GenBank. The posterior probability values are indicated at nodes.

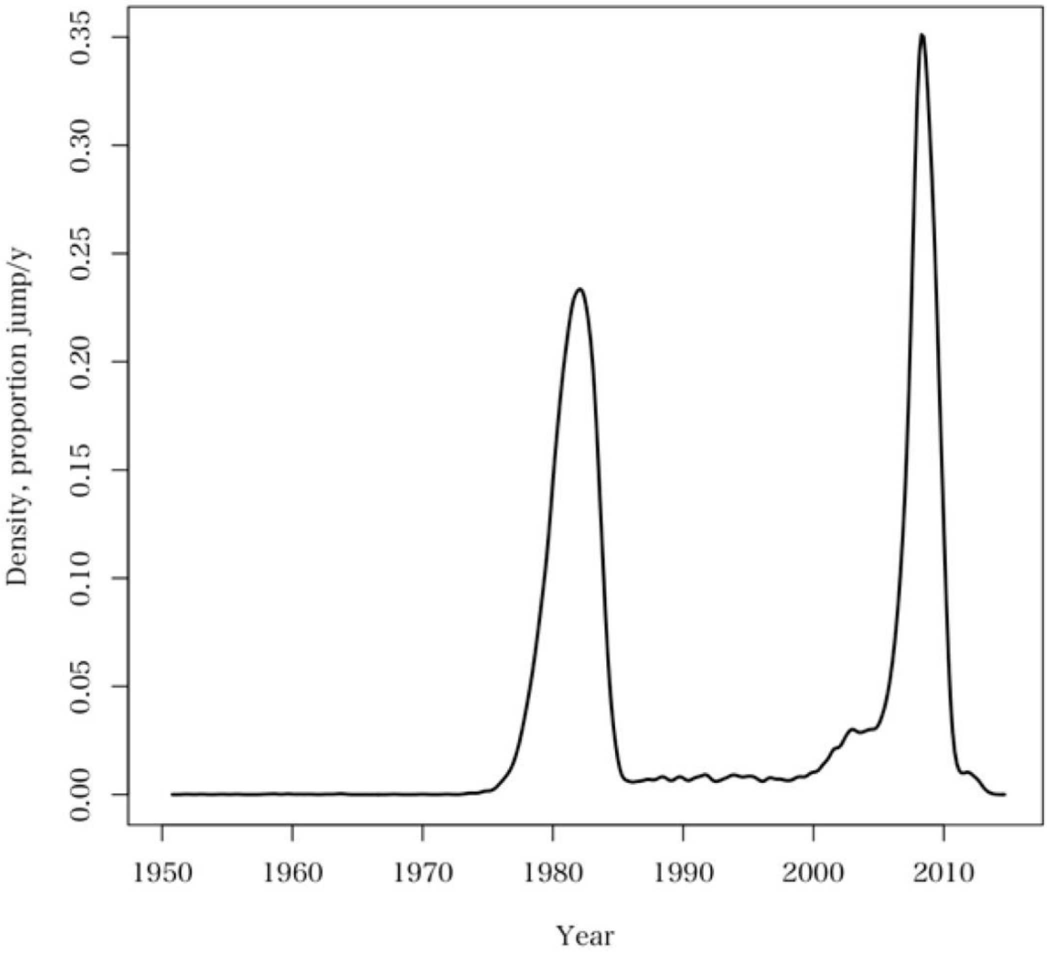

Technical Appendix Figure 2. Markov jump density of viral migrations of chikungunya virus over time between Philippines and Indonesia. The Markov jump along the branches of the posterior trees of Asian genotype were estimated, and the history of the migrations between the Philippines and Indonesia was inferred by summarizing the Markov jump density using kernel density estimation.
